# Transcriptional Regulation of Lineage Commitment - A Stochastic Model of Cell Fate Decisions

**DOI:** 10.1371/journal.pcbi.1003197

**Published:** 2013-08-22

**Authors:** Jose Teles, Cristina Pina, Patrik Edén, Mattias Ohlsson, Tariq Enver, Carsten Peterson

**Affiliations:** 1 Computational Biology & Biological Physics, Department of Astronomy and Theoretical Physics, Lund University, Lund, Sweden; 2 Stem Cell Group, UCL Cancer Institute, University College London, London, United Kingdom; EMBL-European Bioinformatics Institute & Wellcome Trust Sanger Institute, United Kingdom

## Abstract

Molecular mechanisms employed by individual multipotent cells at the point of lineage commitment remain largely uncharacterized. Current paradigms span from instructive to noise-driven mechanisms. Of considerable interest is also whether commitment involves a limited set of genes or the entire transcriptional program, and to what extent gene expression configures multiple trajectories into commitment. Importantly, the transient nature of the commitment transition confounds the experimental capture of committing cells. We develop a computational framework that simulates stochastic commitment events, and affords mechanistic exploration of the fate transition. We use a combined modeling approach guided by gene expression classifier methods that infers a time-series of stochastic commitment events from experimental growth characteristics and gene expression profiling of individual hematopoietic cells captured immediately before and after commitment. We define putative regulators of commitment and probabilistic rules of transition through machine learning methods, and employ clustering and correlation analyses to interrogate gene regulatory interactions in multipotent cells. Against this background, we develop a Monte Carlo time-series stochastic model of transcription where the parameters governing promoter status, mRNA production and mRNA decay in multipotent cells are fitted to experimental static gene expression distributions. Monte Carlo time is converted to physical time using cell culture kinetic data. Probability of commitment in time is a function of gene expression as defined by a logistic regression model obtained from experimental single-cell expression data. Our approach should be applicable to similar differentiating systems where single cell data is available. Within our system, we identify robust model solutions for the multipotent population within physiologically reasonable values and explore model predictions with regard to molecular scenarios of entry into commitment. The model suggests distinct dependencies of different commitment-associated genes on mRNA dynamics and promoter activity, which globally influence the probability of lineage commitment.

## Author Summary

Stem cells have the capacity to both self-renew and differentiate into specialized cell lineages, thus sustaining tissue formation during embryonic development and permitting tissue homeostasis throughout adult life. Previous studies have suggested that stem cell commitment to a specific lineage may constitute a discrete event of stochastic activation of a small number of key regulator genes. Experimental exploration of this question is challenging, in face of the elusive nature of the commitment transition and due to considerable gene expression heterogeneity between cells. Here, we implement a computational model that simulates gene expression variation through time and affords the capture of *in silico* commitment events. This model integrates statistical analysis of experimental single-cell gene expression data with dynamical modeling methods to implement a mechanistic framework for stochastic regulation of gene transcription and a probabilistic approach for the commitment rules. Applied to blood cells, our method identifies potential commitment-associated genes, explores how their expression patterns can define alternative commitment regimes, and suggests how differences in regulation of gene expression dynamics can impact the frequency of commitment.

## Introduction

Understanding how primary stem and multipotent progenitor cells decide their fate is pivotal in studying mechanisms driving tissue development and maintenance in multicellular organisms. Despite considerable advances in ascribing key genes and regulatory circuits to specific lineages, the diversity of molecular mechanisms employed by individual cells to commit to particular lineage fates remains largely uncharacterized. Recent technical developments in quantitative measurements of single-cell gene expression [1], [2] have revealed stem and progenitor cell populations to be highly heterogeneous, and suggest that individual cells can exhibit transient biases towards different lineages, even in clonal populations [3]–[10]. This molecular heterogeneity may result from stochastic fluctuations caused by noisy gene expression [11], leading to fluctuations in individual mRNA molecule transcription and degradation rates, and likewise for protein production in individual cells [12], [13]. Also, genes switch between active and inactive states, alternating between variable-length transcriptional bursts that can produce a large number of mRNA molecules, and refractory periods in which transcription is significantly reduced [14], [15]. Molecular mechanisms of commitment have been suggested to involve various degrees of gene expression coordination, from activation of a few genes [16] to gradual accumulation of a transcriptome-wide coordinated program [17]. Finally, the role of external cues (e.g. growth factors) in commitment remains unresolved, with a long-standing debate on whether they can instruct cells to commit to a particular fate, or do merely act as survival factors of cells that have committed through intrinsic mechanisms [18], [19]. A considerable hurdle in elucidating these questions is the elusive nature of the lineage commitment transition, which confounds the experimental capture of cells undergoing commitment. Recent advances in microscopy and imaging techniques enabled the tracking of single cells in time [20]. However, the ability of such methods to simultaneously track expression of multiple genes at the single molecule level is still limited, more so for endogenous genes, which may have a role in effecting commitment decisions [2]. Additionally, the molecular heterogeneity of individual committed cells poses a challenge for defining the relative contributions of single regulators, both individually and in combination, to transitions.

In this work we follow an integrative approach aiming at computationally modeling the stochastic dynamics of lineage commitment of individual multipotent progenitor cells. We do so using static gene expression profiles of individual self-renewing (SR), erythroid-committed progenitors (CP) and erythroid-differentiated (Ediff) cells, obtained from the bone marrow-derived multipotent hematopoietic cell line EML, for a panel of genes putatively relevant for erythroid and myeloid lineage development (Methods) [21].

**Figure 1:**
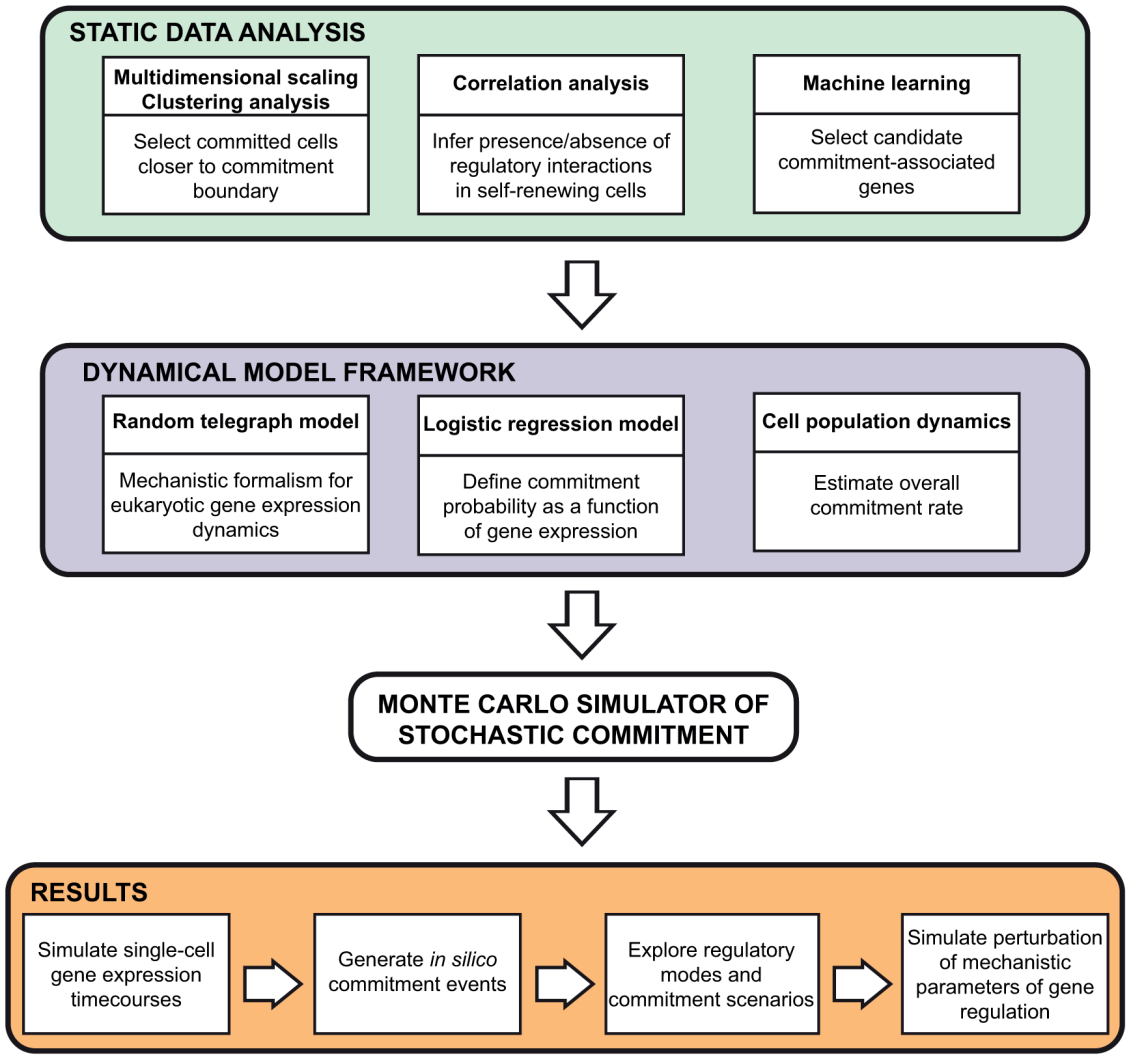
Computational approach. *Top panel:* Static data analysis allows inference of system-specific features: clustering analysis and multidimensional scaling (dimensionality reduction method) delineate a population of progenitors closer to commitment boundary; pairwise gene expression correlation analysis assesses presence or absence of regulatory interactions in self-renewing progenitors; machine learning methods identify candidate commitment-associated genes. *Middle panel:* The dynamical model framework is set by three fundamental components: the random telegraph model is used as the mechanistic formalism for the non-poissonian behavior of eukaryotic gene expression dynamics; a logistic regression model trained with single cell data defines the probability of commitment as a function of gene expression; a cell population dynamics model allows estimation of the overall commitment rate from culture data. *Bottom panels:* We implement a Monte Carlo simulator of stochastic commitment that integrates the static data analysis with the dynamical model framework. The simulator generates single-cell expression time-courses for multiple genes simultaneously and captures *in silico* commitment events. Statistical exploration of the gene expression patterns underlying these events allows the characterization of gene-specific regulatory modes and their influence in the probability and frequency of commitment.

We first perform an exploratory analysis of the static gene expression data, which provides insight into relevant features of the multipotent and committed progenitor populations as well as the SR-to-CP transition (Figure 1 - top panel):

- Clustering methods identify CP cells closest to the commitment boundary.
- Pairwise gene expression correlation analysis assesses the presence/absence of gene regulatory interactions.
- Machine learning methods identify putative commitment-associated genes and formalize probabilistic rules of commitment.

Based upon these results, we implement a novel expansion of the random telegraph model of transcriptional bursting [15], [22] that provides a framework for stochastic commitment as a function of mechanistic aspects of gene expression dynamics (Figure 1 - middle panel):

- The parameters of the generated expression time series are fitted to static expression data of key commitment-associated genes in SR cells, providing a mechanistic framework for the non-Poissonian gene expression behavior observed in eukaryotic cells.
- A logistic regression model trained with SR and CP expression data defines the probability of commitment in time as a function of the simulated gene expression profiles.
- Modeling of SR and CP cell culture dynamics data allows inference of a characteristic time of commitment, providing a link between simulated Monte Carlo and physical times.

This integrative approach is based on, and expands upon, recently published single cell expression data from the hematopoietic EML cell line for populations in the vicinity of the erythroid commitment boundary [21]. We revisit the question of transcriptional program coordination at the outset of lineage specification through correlation analysis and infer putative regulators of the commitment transition. Additionally, we explore the regimens of transcriptional regulation for these genes in the context of a stochastic model of transcriptional bursting and implement expression-dependent rates of commitment which allow the capture of simulated cells at the moment of transition and the assessment of how mechanistic parameters of gene expression regulation impact on the frequency of commitment (Figure 1 - bottom panel).

## Results

### The transition into commitment - static data analysis

The single-cell expression data in [21] is a valuable resource for studying the regulation of commitment transitions as it captures SR and CP cells in direct ontogenic relationship. Of note, CP cells represent a uniquely early stage post-commitment but are also more molecularly heterogeneous. In order to focus on molecular programs at the commitment transition boundary, we used a combination of hierarchical clustering and dimensionality reduction methods to identify sub-compartments amongst CP cells (Figure S1, Methods). We isolated a minor subset of cells (CP2) that are apparently late in their expression profiles and cluster with Ediff cells. The remaining CP cells, denoted CP1, are distinct from SR and Ediff and could not be further subdivided, and are thus used as early-committed CP cells in what follows.

We compared the frequency and level of expression of all 17 individual genes (Text S1) in each of the compartments SR, CP1, CP2 and Ediff (Figure S2). A set of genes displays monotonic increase in frequency and/or average level of expression from SR through Ediff (e.g. *Gata1*); the converse monotonic trend is observed for a smaller set of genes (e.g. *Mpo*). Interestingly, other genes have non-monotonic patterns of expression increasing at the SR to CP1 transition, to then decrease during differentiation (e.g. *Gata2*), or decreasing from SR to CP2, to increase in the Ediff compartment (e.g. *Btg2*). Pronounced changes between cell types can suggest functional relevance in commitment and/or differentiation.

We then calculated pairwise Spearman correlations for all genes within the SR and CP1 compartments to assess overall coordination of transcriptional programs at the commitment transition (Figure S3, Tables S1, S2, S3, Methods). Despite the choice of an inclusive correlation coefficient cutoff value, SR cells did not show broad gene-to-gene correlation. Similarly, gene expression in the CP1 population is essentially uncorrelated, with a low number of weak correlations. In contrast, a highly correlated and interconnected gene network could be observed for Ediff cells. Of note, *Gata1* and *Epor*, which are critical regulators of erythroid lineage development, are minimally or not at all correlated in SR or CP1 compartments. Hence, this analysis shows no evidence of significant gene regulatory interactions around or at the point of erythroid commitment within our dataset, consistent with the findings in [21].

### Expression of Gata2 and Mpo are the best predictors of early committed cells

**Figure 2:**
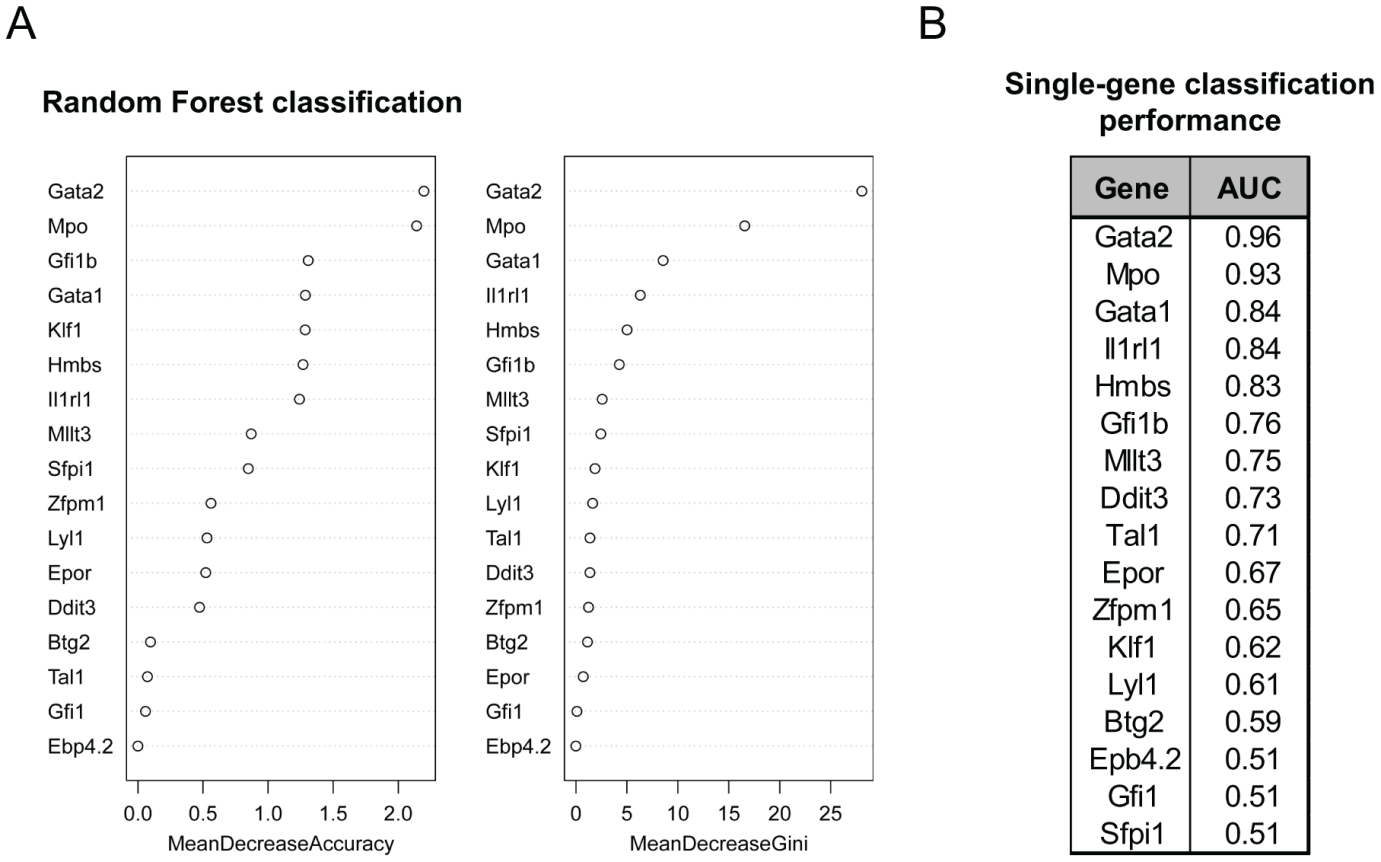
Machine learning methods identify putative commitment-associated genes from the committed progenitor (CP1) versus self-renewing (SR) populations. (A) A random forest classifier (1000 trees and 5 variables per node) was trained to discriminate between the SR and CP1 populations using as input expression data for all genes simultaneously. Variable importance, as measured by the mean decrease in accuracy (left panel) or the Gini coefficient (right panel), was computed using the out-of-bag error. Genes are shown in descending order of importance. (B) Area under the receiving operator characteristic (ROC) curve for individual genes in the data set, measuring the performance in separating between SR and CP1 compartments. *Gata2* and *Mpo* are the top performing genes, measured both by non-linear and linear methods.

We sought to identify the genes that best distinguish between the SR and CP1 populations, which we assume may function directly or indirectly in the commitment transition. Using the single-cell expression data for all genes in both compartments, we first used a random forest classifier [23] (Methods) and evaluated the importance of each gene for the overall performance (Figure 2A). In this analysis, *Gata2* and *Mpo* were by far the most important genes, with *Gata1* ranking at the top of a second line of predictors. Classifier performances are commonly measured by the Receiver Operating Characteristics curve (ROC), which provides performance percentages for different discrimination thresholds. The areas under the ROC curve (AUC), which measure the ability of each gene on its own to discriminate between the two populations (1 being perfect and 0.5 no better than random), are shown in Figure 2B. Again, *Gata2* and *Mpo* ranked highest, with *Gata1* following at the top of a second line of predictors. The random forest classifier covers both linear and non-linear relations between the input variables (in our case gene expressions) and the output class, where linearity represents the weighted sum of the inputs and non-linearity encompasses more complicated relations (e.g. combinations of products). To investigate the presence of the latter we then explored an artificial neural networks (ANN) classifier using *Gata1*, *Gata2* and *Mpo* expressions as inputs varying the number of hidden nodes (Methods). We did not observe a difference in validation performance when comparing non-linear and linear methods, suggesting the absence of more complex relations between the genes. In other words, for the genes in our dataset, the transition from SR to CP seems to be dominated by independent expression values adding up to a certain threshold with gene-specific weights set by the classifier (Methods). Furthermore, to confirm the dominance of *Gata2* and *Mpo* when predicting the commitment probability, we trained ANN models with fixed complexity, using all possible combinations of one up to four genes as inputs. Consistently with our observations, all combinations with the highest cross validation performance included *Gata2* and *Mpo* (data not shown).

Regarding the biological relevance of the three top performing genes, *Gata2* is required for development of the blood system [24], [25], and regulates the adult stem cell compartment through effects on cell cycle [26], [27]. *Mpo* expression can be detected in multipotent as well as myeloid-restricted cells [28], [29]. It constitutes a regulatory hub on which transcription factors such as *Runx1*, *Pu.1* and members of the *Cebp* family converge [30], [31]. *Gata1* is a master regulator of erythropoiesis capable of reprogramming to the erythroid lineage [32], [33], although its requirement in the commitment decision remains unclear [34], [35].

### Stochastic modeling provides mechanistic insight into modes of gene expression regulation in commitment-relevant genes

**Figure 3:**
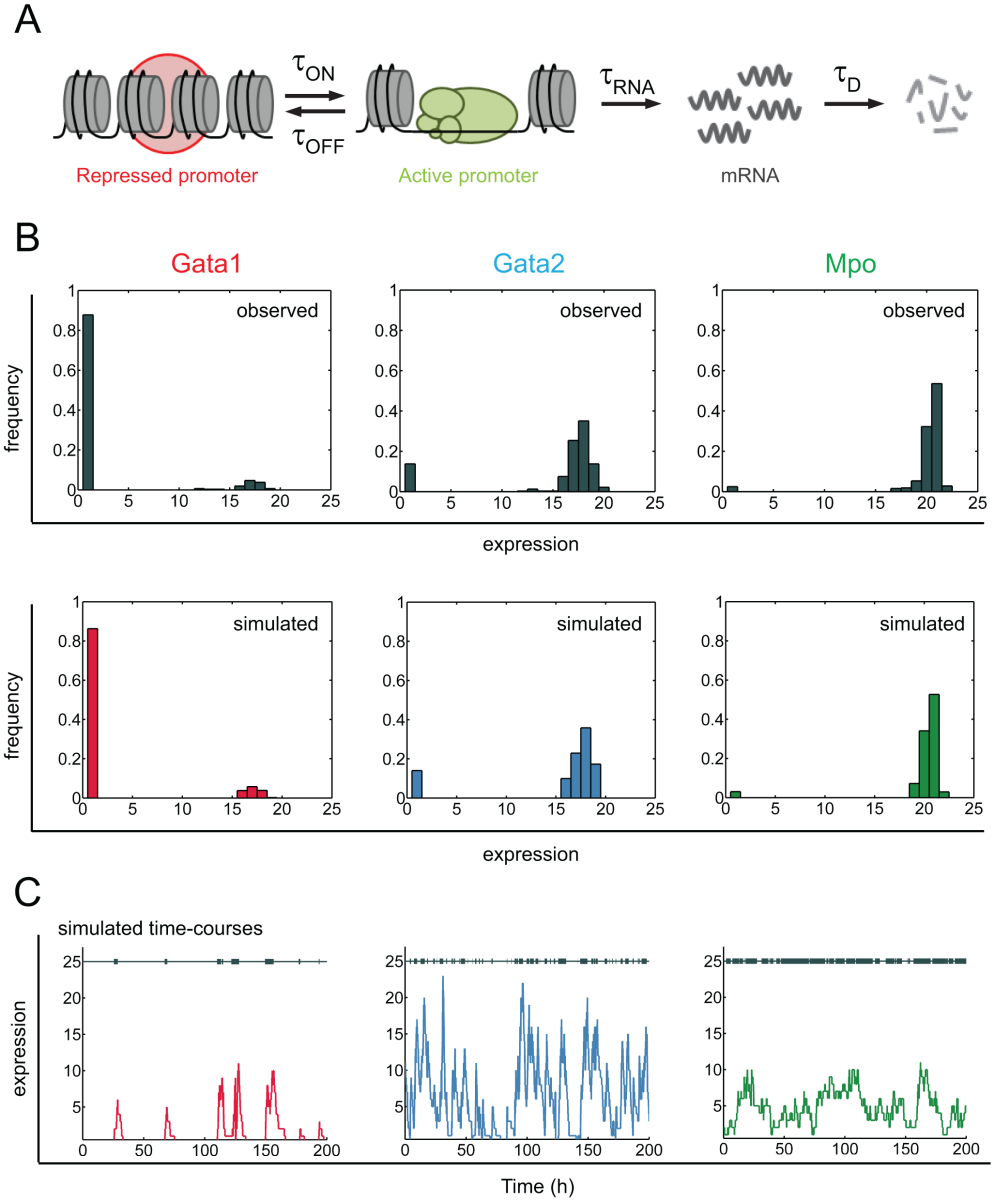
Stochastic modeling of gene expression suggests different modes of regulation for relevant genes. (A) Schematic representation of the random telegraph model for transcriptional bursting. For a given gene, the promoter can be in two different states, active or repressed, with the average time spent in each state being controlled by the average times for activation (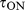) and repression (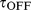). When in the active promoter state, the gene is transcribed and produces mRNA molecules after an average production time 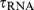. Finally, mRNA molecules are degraded after an average time, 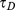, irrespective of promoter states. (B) Best parameter sets for each gene allow for the reconstitution of the experimentally observed distributions (top) within our model simulations (bottom). (C) The parameters suggest different modes of stochastic expression for the different genes, with highly variable burst frequencies and duration (grey bars) as well as mRNA dynamics (colored lines).

**Figure 4:**
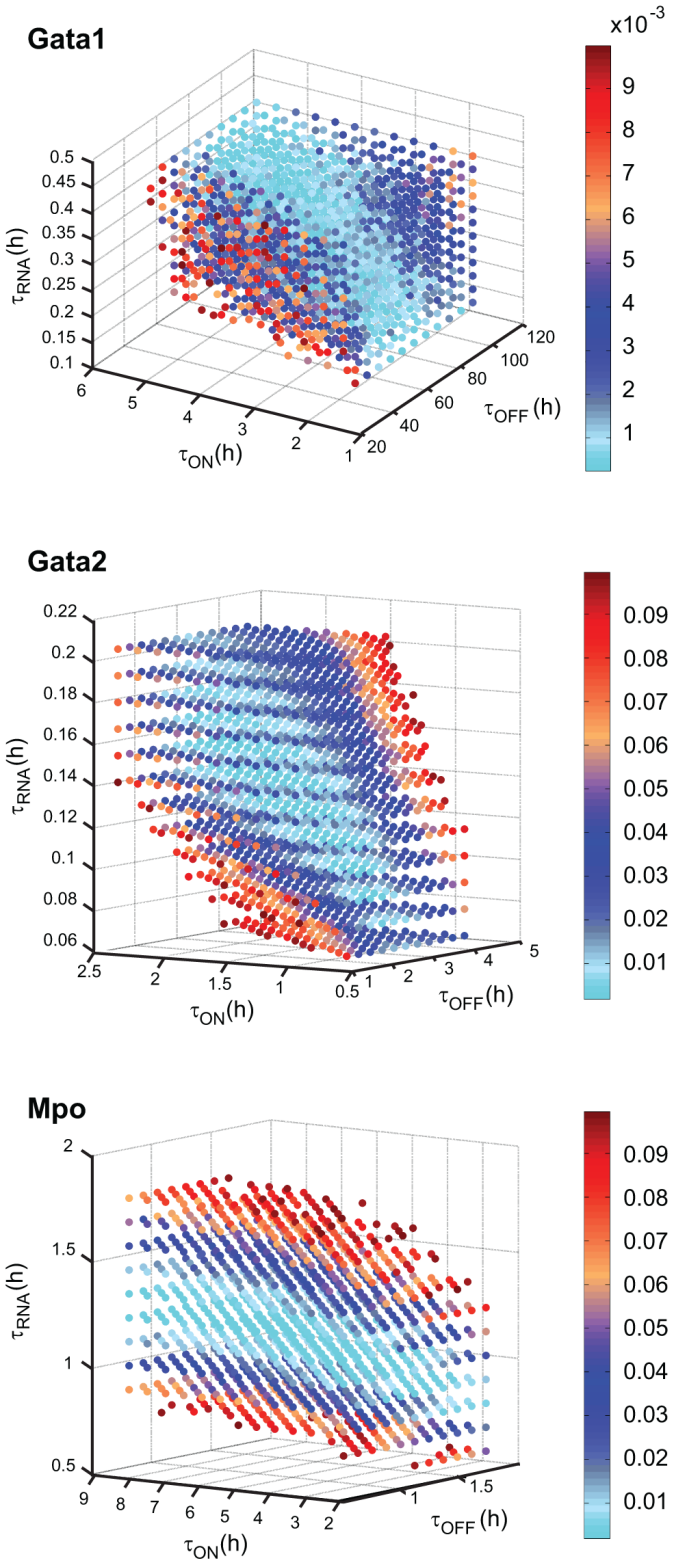
Robustness of the best parameter sets for the random telegraph model. Values for the 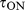, 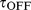 and 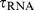 parameters in each gene were varied from 0.5 to 1.6 times the optimum value (in 0.1 intervals); 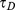 is a fixed parameter in the model and was not varied in this analysis. Within this range, the summed squared error was calculated for all possible parameter combinations in each gene (color scale). For clarity, only solutions below a set error cutoff are represented. Errors calculated for 15000 hours of Gillespie time.

In order to explore the stochastic dynamics of gene expression for the putative key commitment-associated genes, we have used a random telegraph stochastic model for transcriptional bursting [15], [22] (Methods), which provides a mechanistic framework for the non-Poissonian behavior observed in eukaryotic gene expression (Figure 3A). Considering our previous results, we followed a consensus approach and selected genes that consistently ranked high in all classification methods: *Gata2* and *Mpo* were the two best predictors of the committed state and *Gata1*, which also ranked consistently high, is well-described as a master regulator of erythroid differentiation capable of myeloid and lymphoid cell reprogramming to an erythroid fate, making it a likely candidate driver of erythroid commitment. These three genes have distinct gene expression profiles in SR cells, providing an opportunity to assess how distinct modes of gene regulation can affect fate transitions. We fitted model parameters for each of the three genes through simulated annealing, followed by grid search optimization, minimizing the error towards the experimentally observed distributions (Figure 3B). The mRNA decay parameter was fixed for each gene according to published data [36], [37]. The best parameter sets reproduce experimental distributions and provide insight into the gene-specific stochastic dynamics of expression, suggesting that the three genes have distinct modes of regulation (Figure 3C, Table S4). *Gata1* displays short infrequent bursts of transcriptional activity; *Gata2* expression is set by short but frequent transcriptional bursts with high mRNA production rate; *Mpo* is expressed through very long bursts of promoter activity resulting in near-constitutive expression. We tested the robustness of these parameter sets by exploring different combinations of parameters in the vicinity of the optimum solutions (Figure 4, Methods). Given its low frequency of expression, the *Gata1* distribution can be reconstituted by a fairly broad range of parameters and sensitivity is highest to parameters governing promoter activity. In contrast, the parameter space for *Gata2* is constrained to a smaller region around optimum values, with a clear positive correlation between mRNA production and promoter inactivation times. Finally, for *Mpo* the most important parameter is mRNA production time, with a very narrow region of tolerance around the optimal value. Overall, these results suggest that the observed gene expression distributions for the three genes may be governed by different regulatory mechanisms: *Gata1* primarily by promoter activity, *Mpo* primarily by mRNA dynamics and *Gata2* by both.

### Expansion of the stochastic model includes expression-dependent commitment events

**Figure 5:**
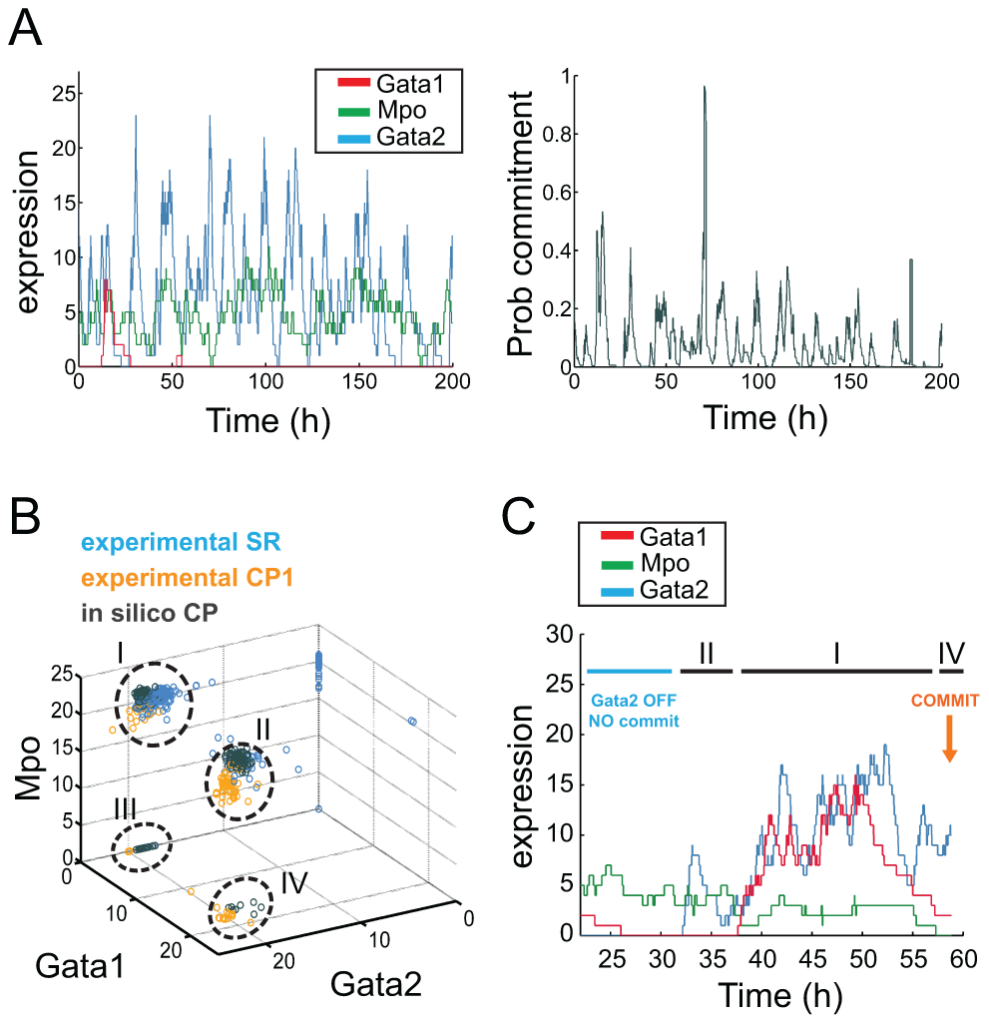
Expression of *Gata2* defines regions of high and low commitment probability. (A) Simulated gene expression time-course for *Gata1* (red), *Gata2* (blue) and *Mpo* (green) and corresponding probability of commitment (grey). Probability of commitment is very low for most of the time-course, punctuated with high probability peaks for specific gene expression combinations. (B) *Gata1*, *Gata2* and *Mpo* expressions of 160 simulated cells at the moment of commitment transition (*in silico* CP, grey) compared to expressions in experimental self-renewing (experimental SR, blue) and committed progenitor cells (experimental CP1, yellow). Absence of *Gata2* expression defines a commitment-impeded region where no commitment events could be observed either experimentally or in simulations; expression of *Gata2* defines a commitment-permissive region where commitment can happen through multiple gene expression combinations: *Gata2* ON / *Gata1* ON / *Mpo* ON (I), *Gata2* ON / *Gata1* OFF / *Mpo* ON (II), *Gata2* ON / *Gata1* OFF / *Mpo* OFF (III), *Gata2* ON / *Gata1* ON / *Mpo* OFF (IV). Instances of *in silico* commitment events through each scenario are presented in Figure S4. (C) Simulated gene expression profile leading into a commitment event: the commitment-impeded region is initially visited (*Gata2* OFF), followed by different combinations within the commitment-permissive region (sequentially II, I and IV), with commitment ultimately taking place with *Gata2* ON / *Gata1* ON / *Mpo* OFF.

We selected the best set of parameters that describe the stochastic dynamics of expression for each of the three genes, and expanded upon the initial model to take into account the probability of a cell to commit as a function of gene expression. Our stochastic model includes an expression-specific commitment rate, proportional to the probability of commitment (Methods). This probability is given by an expression-dependent logistic regression model trained with experimental data, that separates SR from CP populations. The proportionality constant was set to reproduce the average commitment rate inferred from culture reconstitution assays. The logistic regression model captures all relationships between genes, given that non-linear relationships seem to be absent (see classifier analysis above). This simple model for commitment focuses on the experimental data and abstracts the underlying complexity, weighing the importance of individual genes, as well as their combined effects. Since we could not find significant correlations within the SR population suggesting regulatory interactions, we assumed complete independence in the stochastic dynamics of each gene. For most gene expression combinations, the corresponding commitment probability is low, consistent with the fact that commitment is a rare event (Figure 5A). However, for a small subset of expression states, the probability increases sharply. Due to the stochastic nature of the system, we can still observe instances where high probabilities do not lead to commitment, as well as others where commitment happens despite low probabilities.

### Stochastic modeling of commitment highlights individual gene contributions and predicts the outcome of gene expression perturbations

Our modeling approach generated a population of *in silico*-committed cells, and we compared their expression of *Gata1*, *Gata2* and *Mpo* at the moment of transition against experimentally observed values in SR and CP1 cells (Figure 5B). *In silico* CP cells are located at the edge of the SR population and share some characteristics with experimental CP1. In particular, simulated CP cells can recapitulate expression patterns specific to experimental CP1 and absent from SR cells, such as absence of *Mpo* in the presence of *Gata1* and *Gata2*. Events of *in silico* commitment occur more often with high values of *Gata2* and *Gata1*, and indeed, absence of *Gata2* does not seem compatible with CP status. Nevertheless, cells can commit both experimentally and *in silico* with low levels of *Gata2* and in the presence of *Mpo*, if *Gata1* is also present. Given the stochastic nature of the commitment transition, it is possible for cells with commitment-permissive expression profiles not to effect commitment (Figure 5C). It is also possible for cells to commit as soon as they enter a commitment-permissive state, and to do so with different kinetics (Figure S4). Overall, the data are compatible with the existence of multiple transcriptional routes into lineage commitment.

**Figure 6:**
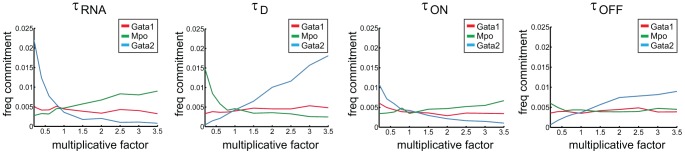
Changes in regulation of *Gata2* at the mRNA level have the strongest impact in overall commitment frequency. Perturbation of stochastic gene expression regulation parameters: values for 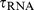, 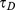, 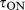 and 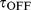 were varied from 0.2 to 3.5 times the optimum values, one gene at a time. Frequency of commitment defined as the number of commitment events per hour of Gillespie time. Each simulation was run for 30000 hours of Gillespie time.

We assessed how graded changes in the parameters governing gene expression regimens affect the frequency of transition to the committed state (Figure 6). Strongest effects are observed upon perturbation of mRNA processing parameters (production and decay), particularly for *Gata2*, whereas similar perturbations at the level of promoter activity state do not seem to cause major commitment frequency changes. This suggests that for *Gata2* as for other putative regulators of lineage commitment with similar expression profiles, mRNA dynamics may play a more important role than the regulation of promoter status (e.g. through histone modifications) in influencing the commitment transition.

**Figure 7:**
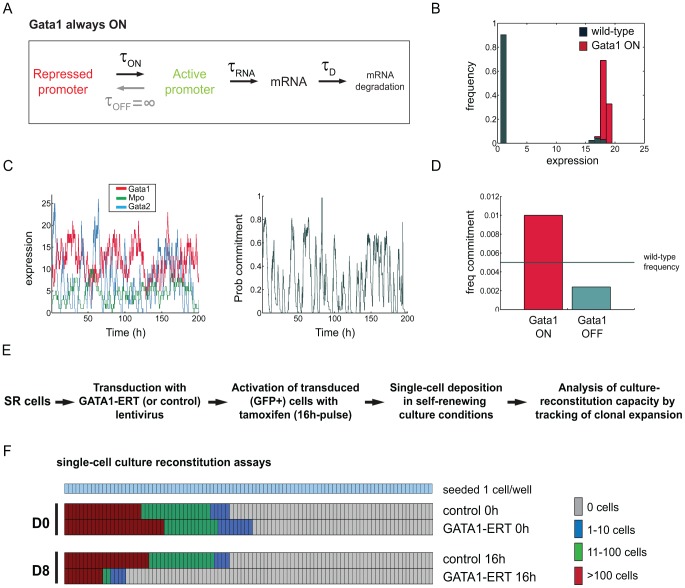
Perturbation of *Gata1* regulatory dynamics impacts frequency of commitment *in silico* and *in vitro*. (A) Simulated *Gata1* regulatory regimen corresponding to permanent activity of the locus. (B) *Gata1* gene expression distribution under simulated expression regimen in A (*Gata1* ON - red); simulated *Gata1* expression in normal conditions (Figure 3B) is presented for comparison purposes (wild type - grey). (C) Simulated gene expression time-course for *Gata1* (red), *Gata2* (blue) and *Mpo* (green) when *Gata1* promoter is permanently in the active state (left panel); the right panel depicts the corresponding probability of commitment. High probability of commitment peaks are more frequent than in wild type simulations (Figure 5A). (D) *In silico* predictions of changes in commitment frequency resulting from permanent activity (ON, red bar) or permanent inactivation (OFF, blue bar) of the *Gata1* promoter. Two-fold changes in the frequency of commitment were predicted. Frequency of commitment defined as the number of commitment events per hour of Gillespie time. Each simulation run for 20000 hours of Gillespie time. (E) Experimental design of GATA1-ERT activation in EML SR cells, mimicking *Gata1* ON conditions. Functional readout is the culture-reconstituting capacity of individual cells washed and cultured after a 16-hour pulse of tamoxifen. Culture-reconstituting cells originate large 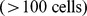 clones [21]. (F) Inspection of clonal culture-reconstitution capacity of transduced EML cells before and after treatment. The 2-fold decrease in large reconstituting colonies between control and GATA1-ERT pulsed cells (red bars) matches the 2-fold gain in commitment predicted in D.

Such subtle changes at this level of gene regulatory mechanisms are seldom feasible in a tightly controlled manner within experimental settings. Instead, gain- or loss-of-function experiments are more often used to assess the functional relevance of a given gene, involving much more pronounced expression increase or decrease, respectively. In this context, we used our stochastic model to predict the impact of pronounced *Gata1* expression changes in the frequency of commitment in the EML model cell system. Despite *Gata1*'s capacity to reprogram cells to an erythroid fate through ectopic expression under a strong exogenous promoter [32], [33], our model suggested a less prominent though relevant role under its native expression regime, and we wished to test the consequences of enforcing its expression both *in silico* and *in vitro*. To this end, we set the 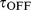 parameter for *Gata1* to an infinite value, thus effectively keeping its promoter permanently in the active state (Figure 7A). The range of simulated values for *Gata1* expression in this perturbation scenario is comparable to wild type, but the fraction of high-expressing cells is greatly increased (Figure 7B). The gene expression time-course reflects the permanent activity of the *Gata1* promoter resulting in more frequent high commitment probability peaks as compared to wild type (Figure 7C and Figure 5A). These changes result in a 2-fold predicted increase in frequency of commitment from wild type to the *Gata1* ON perturbation (Figure 7D). In order to test these results experimentally, we transduced EML SR cells with a GATA1-ERT fusion construct [32], activated the resulting protein with a pulse of tamoxifen (Methods), and assessed the status of the activated cells in clonal culture-reconstituting assays (Figure 7E). Importantly, we were able to recapitulate the 2-fold increase in commitment predicted by our model (Figure 7F).

Overall, the data supports the *in silico* predictions of our stochastic model of commitment and attests to its utility in exploring alternative expression regimens at the transition between self-renewal and lineage commitment.

## Discussion

Our stochastic Monte Carlo model approach is to our knowledge novel. It integrates the random telegraph model framework [15], [22] with commitment probabilities obtained from single cell classifiers and cell culture properties. Also, the robust conversion of static expression data, where each data point is considered a “snapshot”, into time series parameters is new in this context. In [38] cell cycle FISH data were analyzed with the same goal using template matching. Our approach, which can be expanded to a larger number of genes and extended to instances where regulatory interactions are present, provides insight into the mechanistic aspects underlying stochastic gene expression and, more importantly, establishes a link between such mechanisms and functional properties of individual cells, by assessing the relevance of promoter and mRNA regulation dynamics in the frequency of commitment.

The computational framework was designed and implemented using single cell expression data observations from different populations of the EML hematopoietic cell line [21]. Clustering analyses distinguished cellular sub-compartmentalization from molecular heterogeneity within the CP population and identified subsets of early (CP1) and late (CP2) committed cells, with distinct molecular profiles. Global characterization of CP1 cells revealed a heterogeneous population dispersed in their individual expression profiles, including absence of known erythroid regulators like *Gata1*, *Klf1* or *Epor* in a significant number of cells. Importantly, we observed only few and weak pairwise correlations between genes in CP1 cells, a pattern that was even more evident amongst SR cells. Hence, no significant level of gene expression coordination is discernible in the commitment transition, at least not within the gene signature analyzed.

We proceeded to infer potential key commitment regulators using machine learning methods to separate SR from CP1 cells across the commitment boundary. We identified increase in *Gata2* and decrease in *Mpo* expression as the best predictors of commitment, with changes in a second group of genes, including increase in *Gata1* expression, also of some relevance. Although we cannot directly equate predictors of the commitment event with commitment effectors, we have presumed it likely that those genes that best separate SR from CP1 states play a role in their identity or maintenance, and hence may directly effect or report the decision. Also, in exploring mechanisms of commitment, we are aware that our data is exclusively transcriptional and, consequently, mechanistic approaches cannot consider the effects of translational mechanisms and protein quantities. However, protein half-lives for *Gata1* and *Gata2*, for instance, are similar or even shorter than those of their respective mRNAs [36] suggesting that regulation is in fact dominated by transcriptional events. Indeed, short half-lives of both mRNA and proteins seem to be a common feature of genes involved in regulatory mechanisms [39] and partially preclude the existence of buffering effects at the protein level, although they cannot account for all translational regulatory events.

A better understanding of the regimens of expression of *Gata2*, *Mpo* and *Gata1* and their consequences for the SR-to-CP transition could illuminate specific and global mechanisms of lineage commitment. Thus, we explored the dynamics of these three genes by fitting the parameters of a stochastic gene expression model to experimentally observed distributions. These solutions, validated by a local robustness analysis, were taken as strong indicators of the qualitative behavior of the system. We found the genes to have different regulatory dynamics, compatible with global experimental observations in mammalian genes [40]. In the case of *Gata1* and to some degree *Gata2*, the frequency of promoter activity bursts plays a fundamental role; *Mpo*, on the other hand, is most sensitive to variations in mRNA production times. These patterns are consistent with measurements in yeast, in which transcriptional bursts were more important for larger variations, whereas smaller variations were mostly attributed to transcription-initiation mechanisms [41]. We extended the stochastic model to account for commitment events by means of a logistic regression model that maximizes the separation between SR and CP cells; stochastic commitment events were thus the result of (i) the inherent stochasticity resulting from the mechanistic parameters of *Gata1*, *Gata2* and *Mpo* regulation, and (ii) the rate of commitment inferred from SR-seeded cell cultures, itself implemented as a random event. Within this framework, the probability of commitment is very low for the vast majority of the time, with infrequent and short transient peaks at high values. This behavior bears some resemblance to excitable systems of differentiation [42]. The extended model allowed us to recreate and capture *in silico* the moment of commitment. By analyzing the molecular patterns of simulated cells at the transition, we hypothesize that expression of *Gata2* defines two states in SR cells: a commitment-impeded state with low *Gata2* expression in which no commitment events were observed; and a commitment-permissive state with high *Gata2* expression where multiple entry points into commitment can be reached. Given the lack of correlations between the expression of *Gata2* and other genes, we could not further explore specific molecular mechanisms by which *Gata2* can drive cells into commitment. Nevertheless, we systematically assessed how gradual changes in the stochastic dynamics of gene expression regulation for *Gata2*, *Mpo* and *Gata1* influence the frequency of commitment. Again, changes in *Gata2* regulation had the strongest impact, in particular when perturbing mRNA production and decay. Additionally, we tested the impact of more drastic changes in regulatory parameters, by simulating permanent activity of the *Gata1* promoter. The predicted 2-fold increase in frequency of commitment is in agreement with experimental results measuring loss of culture-reconstitution capacity in clonal assays, and is compatible with the reported role of *Gata1* in erythroid differentiation and reprogramming experiments [32], [33], [35]. Taken together, these observations bridge mechanisms of gene regulation and functional impact on lineage commitment, and highlight the role of intrinsic noise in cell fate decisions [43]. This integrative approach can also be applied to other differentiating systems, generating hypotheses on transcriptional regulation dynamics and its impact on commitment.

## Methods

Single cell expression, clonal culture-reconstitution and *Gata1* perturbation data are described in Text S1.

### Relating gene expression values with multiplicities

The gene expression data (see Text S1) were originally expressed as 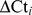 for each gene *i* to reference Atp5a1 and linearly transformed to the variable
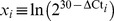
(1)where 30 is the experimental detection limit. The variable 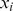 grows with multiplicity in contrast to 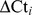. To confront modeled distributions of multiplicities 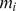 with measured 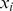-distributions, we assumed
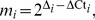
(2)where 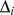 is a gene specific parameter. This represents an ideal experiment, where abundances double in every amplification cycle, and a single molecule is eventually detected after 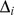 cycles. The threshold 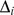 may be gene specific, depending on properties of the reference reporter used. Thus, we get
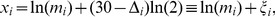
(3)where 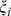 is a gene specific shift parameter to be fitted together with the model rates (Table S4). We should stress that single-cell RT-qPCR data is a relative measure of mRNA abundance for each individual gene analyzed. Quantification is obtained by measuring the number of amplification cycles needed to detect individual mRNA species above an experimental threshold. This detection threshold may represent a different number of mRNA molecules for each gene, since the measured relative level depends on gene-specific parameters (such as amplification efficiency from the initial mRNA molecule number) as well as on the interrogating primers/probe. As a consequence, comparisons of single-cell expression levels are internally consistent and can be made between populations for a given gene (such as presented in Figure S2) but do not reliably measure differences between genes in a given population. The shift parameter, 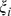, takes into account gene-specific detection thresholds and unique amplification efficiencies, mapping the number of mRNA molecules in our Monte Carlo simulations onto the experimentally-observed gene expression scale.

### Data mining and classifiers

#### Clustering analysis

Hierarchical clustering of single cells was performed using Euclidean distance and complete linkage. Expression values were mean-centered and divided by standard deviation for each cell. The analysis was performed with Genesis [44].

#### Dimensionality reduction analysis

Multidimensional scaling (MDS) was used to visualize the relative position of single cells in the different populations, based on their individual gene expression profiles, reducing the 17-dimensional space (one per gene) to two-dimension representations. For this purpose, MDS provides similar results compared to other dimensionality reduction methods such as Principal Components Analysis. MDS performed using the Statistical Toolbox on Matlab (MathWorks).

#### Correlation analysis

Spearman rank correlations were calculated between all pairs of genes co-expressed by a minimum of 10 cells in the population. Due to the limited amount of available data and the relative novelty of the approach, the choice of an optimal significance cut-off for defining pairwise correlations using single-cell gene expressions remains experimental. Our main goal was to broadly characterize the regulatory potential around or at the point of commitment, so we opted for an inclusive approach and considered as significant, correlations with coefficient values above 0.3 at a 99% significance level. This choice of cutoff is supported by recent literature [45], and the significance level corresponds to a 0.01 probability of having a correlation as large or higher than the observed value, by chance, when the true correlation is zero. Calculations were performed using the Statistical Toolbox on Matlab (MathWorks). Interaction plots based on significant correlations for SR, CP1 and Ediff were produced using Cytoscape [46].

#### Prediction models

A random forest classifier [23] was trained to distinguish between SR and CP populations using single cell expression data from all genes.The random forest method consists of a collection of fully trained decision trees. It can be considered as an ensemble learning method for classification problems that combines a random selection of both data (bagging [47]) and features.Our random forest model used 5 variables at each node and 1000 trees. Permutation variable importance and Gini coefficients were computed using the out-of-bag error [23] and used to rank the most important genes. All runs were made using the random forest R package.

Logistic regression linear classifiers were used to infer best commitment predictor genes, as well as to provide commitment probabilities (i.e. transition from SR to CP compartment) as a function of gene expression. The classifiers were trained to separate SR and CP1 populations, using both single gene and multiple gene measurements as inputs. We used the logistic regression model to calculate the probability of commitment according to,
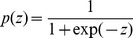
(4)and
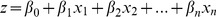
(5)Here 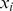 are the expression values for each gene and 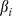 are the regression coefficients quantifying their relative importance, and determined during training.Performance was measured using the area under the receiver operator characteristic (ROC) curve. All calculations were performed using the Statistical toolbox in Matlab (MathWorks).

Artificial neural networks (ANN) (see e.g. [48]) classifier models were used to investigate possible complex relations between gene expression values when comparing the SR and CP1 populations, Each ANN model consisted of an ensemble of multilayer perceptrons with one hidden layer, each trained using gradient descent on a cross entropy error function. The ensemble was constructed using the Bagging method [47] with a fixed size of 20 networks. To assess the performance we used 5-fold cross validation, repeated 20 times each with different splits of the data set. Possible complex relations between input variables can be detected if ANN models with more than one hidden node results in higher validation performance as compared to the (linear) logistic regression model.

### Time evolution - the Monte Carlo model

Time evolution is performed using the Gillespie MC algorithm [49] on the random telegraph model for transcriptional bursting [14], [22]. A given gene *i* is defined by its promoter state 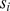


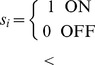
(6)and multiplicity 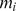. Different actions *a* can take place:

- changing promotor state (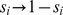)
- production of a mRNA molecule (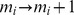)
- decay of a mRNA molecule (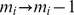)
- commitment to the CP state.

We pick times 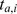 for potential actions *a* for each gene *i* from exponential distributions
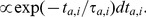
(7)where the 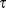-parameters are 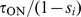 for turning the promotor on, 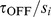 for turning it off, 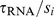 and 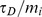 for production and decay of mRNA respectively.

With 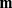 representing the different components 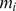, we use 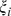 (Eq. 3) and the trained logistic regression classifier (Eqs. 4 and 5) to calculate the state-dependent commitment rate 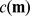 as explained with Eq. 16 and pick a time for potential commitment, with the 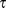 parameter 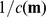. Optimized parameter values are found in Table S4.

The action with the shortest time is selected and the time spent in the current state is recorded. Then the state is updated and new times are selected. After completed simulation, the fraction of time spent in a state is our resulting probability for finding a cell in that state. The system is thermalized for each new cell by requiring the promotor to turn ON and OFF at least once for each gene.

### Determining the commitment rate from population dynamics

The dimensionless time scale of the Monte Carlo procedure is related to physical time by inferring the characteristic time of commitment events as a function of gene expression. This is accomplished in two steps: i) the overall commitment rate is inferred through the implementation of a compartmental model describing the dynamics of SR and CP cultures in time, where parameters of cell division and death are fitted to experimental SR and CP cell culture dynamics data ; and ii) the expression-specific commitment rate is obtained by combining overall commitment rate with the commitment probabilities given by the logistic regression classifier for a finite set of genes (Eqs. 4 and 5).

#### Population model

Independently of other cells, each SR cell is assumed to divide with a rate *s*, commit to CP with a rate *c*, and die with a rate *d*. Similarly, CP cells divide and die with rates *s′* and *d′* (Figure S5A).

With *R* and *P* as the number of SR and CP cells, respectively, the evolution equations read
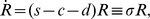


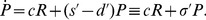
(8)Given initial contrations 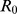 and 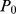, and the parameter
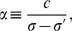
(9)the solutions read
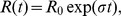


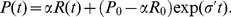
(10)


Provided 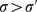, the asymptotic solution is 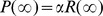. With data suggesting roughly 10% CP after a long time, we get
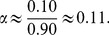
(11)With Eq. 10 and the SR and CP initiated cell culture data (Text S1) we then obtain parameter values in Figure S5B. For each assay, cells were counted after 24 h and 48 h, giving two independent measurements.

In the CP assay, we have 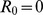, which gives 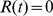 and the simplified equation
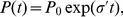
(12)which determines 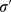. In the SR assay, we do not identify SR and CP cells, but only count the total number of live cells 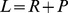. The initial conditions are 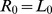 and 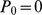. This implies
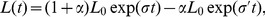
(13)which determines 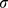 using the 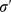 estimate from Eq. 12. From Figure S5B, we note that 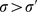 as assumed when estimating 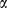 above.

The 24h and 48h results are in excellent agreement for 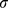 and 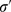 and reasonably so for *c*. Since the 48h data are less vulnerable to statistical fluctuations, we use that time point to determine *c*. Thus, in our Gillespie MC simulations above, we used a commitment rate 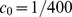 per hour.

The abstract rates 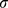 and 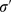, being “net self-renewing rates”, can be translated into division and death rates using data on dead cells. The evolution equation for the number of dead cells, *D*, reads 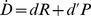. With inital concentration 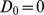, the solution reads
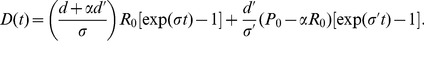
(14)The CP assay conditions 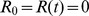 then determines *d′* and 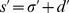, after which the SR assay condition 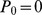 determines *d* and 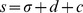.

As interpretation of the numbers in Figure S5B, we note that the average time until division for a SR cell, 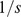, is one day, and that on average 5% of the SR cells commit within this time frame.

#### Expression-specific commitment rates

The probability for a cell to commit is expected to depend on gene expression, which we represented by the vector 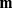 with components 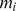 for each gene *i* defined in Eq. 2. In a deterministic model, we would then ask for the time it takes to reach a commitment criteria from different non-committed states 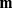, and the probability of being in such a state 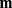 at the beginning of observations.

However, given an expression profile with only a few genes, we must introduce a probability of commitment, reflecting missing information about other genes. We therefore introduce an expression-specific commitment rate, 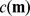, which is high if 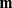 implies high commitment probability, and 0 if 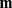 implies 0 commitment probability.

As a simple model, we chose
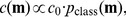
(15)where 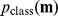 is the classifier probability determined with the logistic regression classifier (Eqs.4 and 5). Furthermore, we assume that expression profiles of the measured genes have thermalized at the beginning of measurements, so that 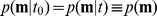.

To give the correct overall commitment rate *c*, the expression-specific rate must be normalized to yield, 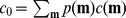. This implies that the expression-specific commitment rate (Eq. 15) is given by
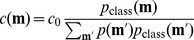
(16)


### Stochastic gene expression model parameter optimization

An in-house implementation of the simulated annealing algorithm [50] was used to optimize parameters for the stochastic gene expression model by minimizing the sum squared error between experimental and observed single-cell gene expression distributions. Optimization was further refined by subsequently performing a local grid search in the vicinity of the best parameter sets.

## Supporting Information

Figure S1 **Molecular characterization of early committed progenitors.** (A) Heatmap of culture-reconstituting, committed progenitor and late erythroid-differentiated cells. A subset of putatively late CP cells clusters together with the Ediff population (red) while the remainder of the CP population forms a heterogeneous but distinct cluster (blue). (B) Expanded view of the cluster in A formed by putatively late CP (yellow) and Ediff cells (red). (C) Multidimensional scaling of all cells based on the expression of the full set of 17 genes. Results confirm the clustering analysis, with SR cells (blue) clearly separated from committed progenitors, which mostly constitute a distinct population (CP1, pink), with the exception of a minority of cells (CP2, orange) that are mixed with terminally differentiated erythroid cells (Ediff, red). (D) Heatmap of committed progenitor cells. The subset of CP cells with a more erythroid-differentiated gene expression signature forms a coherent cluster (CP2, orange), while the remaining cells display more heterogeneous expression profiles (CP1, pink). (E) Multidimensional scaling plot of all committed progenitors shows that CP1 and CP2 cells constitute two distinct populations, with CP1 spreading through a much wider area than CP2, as a result of larger gene expression heterogeneity. Heatmaps were generated by complete hierarchical clustering of individual cells using Euclidian distance; expression values are mean-centered and divided by standard deviation
(EPS)

Figure S2 **Single-cell gene expression profiles.** Single cell level (top) and frequency of expression (bottom) in SR (blue circles), CP1 (yellow diamonds), CP2 (orange squares) and Ediff (red triangles) populations for all genes. Different expression patterns are observed from monotonic increase (e.g. *Gata1*) or decrease (e.g. *Mpo*), to non-monotonic behavior (e.g *Gata2*, *Btg2*), suggesting potential roles in different stages of lineage specification.
(TIF)

Figure S3 **Pairwise correlation analysis of gene expression data.** Significant pairwise correlations between all genes in SR, CP1 and Ediff populations. For each pairwise comparison where at least 10 cells co-expressed both genes, Spearman correlation was considered significant for values above 0.3 at a 99% significance level (Tables S1, S2, S3).
(EPS)

Figure S4 **Commitment can stochastically be driven by different gene expression patterns and at different times.** *Gata1* (red), *Mpo* (green) and *Gata2* (blue) expression in four instances of commitment, simulated with our Monte Carlo model. Each instance (I–IV) corresponds to a commitment scenario, as described in Figure 5.
(EPS)

Figure S5
**Inference of overall commitment rate from compartmental modeling of cell culture dynamics.** (A) Schematic representation of the compartment model describing the number of cells in the self-renewing (SR) and committed progenitor (CP) populations in time. Division rates represented by *s* (SR) and *s*′ (CP); death rates represented by *d* (SR) and *d*′ (CP); commitment rate represented by *c*. (B) Cell numbers from experimental clonal culture-reconstitution observations (top) were used to analytically infer model parameters (bottom). The parameters 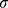 and 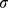
′are defined in Eq. 8 in Methods.(EPS)

Table S1 **Correlation analysis: SR population.** Significant pairwise correlations between all genes in the SR population. For each pairwise comparison where at least 10 cells co-expressed both genes, Spearman correlation coefficient was considered significant for values above 0.3 at a 99% significance level (bold).
(PDF)

Table S2 **Correlation analysis: CP1 population.** Significant pairwise correlations between all genes in the CP1 population. For each pairwise comparison where at least 10 cells co-expressed both genes, Spearman correlation coefficient was considered significant for values above 0.3 at a 99% significance level (bold).
(PDF)

Table S3 **Correlation analysis: Ediff population.** Significant pairwise correlations between all genes in the Ediff population. For each pairwise comparison where at least 10 cells co-expressed both genes, Spearman correlation coefficient was considered significant for values above 0.3 at a 99% significance level (bold).
(PDF)

Table S4
**Parameter values for the random telegraph model of transcriptional bursting (** **Figure 3A** **).** Except for 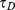, which was obtained from the literature (see Main Text), all parameters were obtained from fitting to experimental SR expression distributions for each gene.(PDF)

Text S1 **Gene expression data, cell culture data and Gata1 perturbation experiments.** The single cell gene expression and clonal culture-reconstitution experiments from [21] are summarized. Details from the Gata1-ERT perturbation experiment are given.
(PDF)
