# Supplementary figures and images for: Transcriptional Regulation of Lineage Commitment - A Stochastic Model of Cell Fate Decisions
Source: PLoS Comput Biol. 2013 Aug 22;9(8):e1003197. doi: 10.1371/journal.pcbi.1003197 (PMC3749951; doi:10.1371/journal.pcbi.1003197)

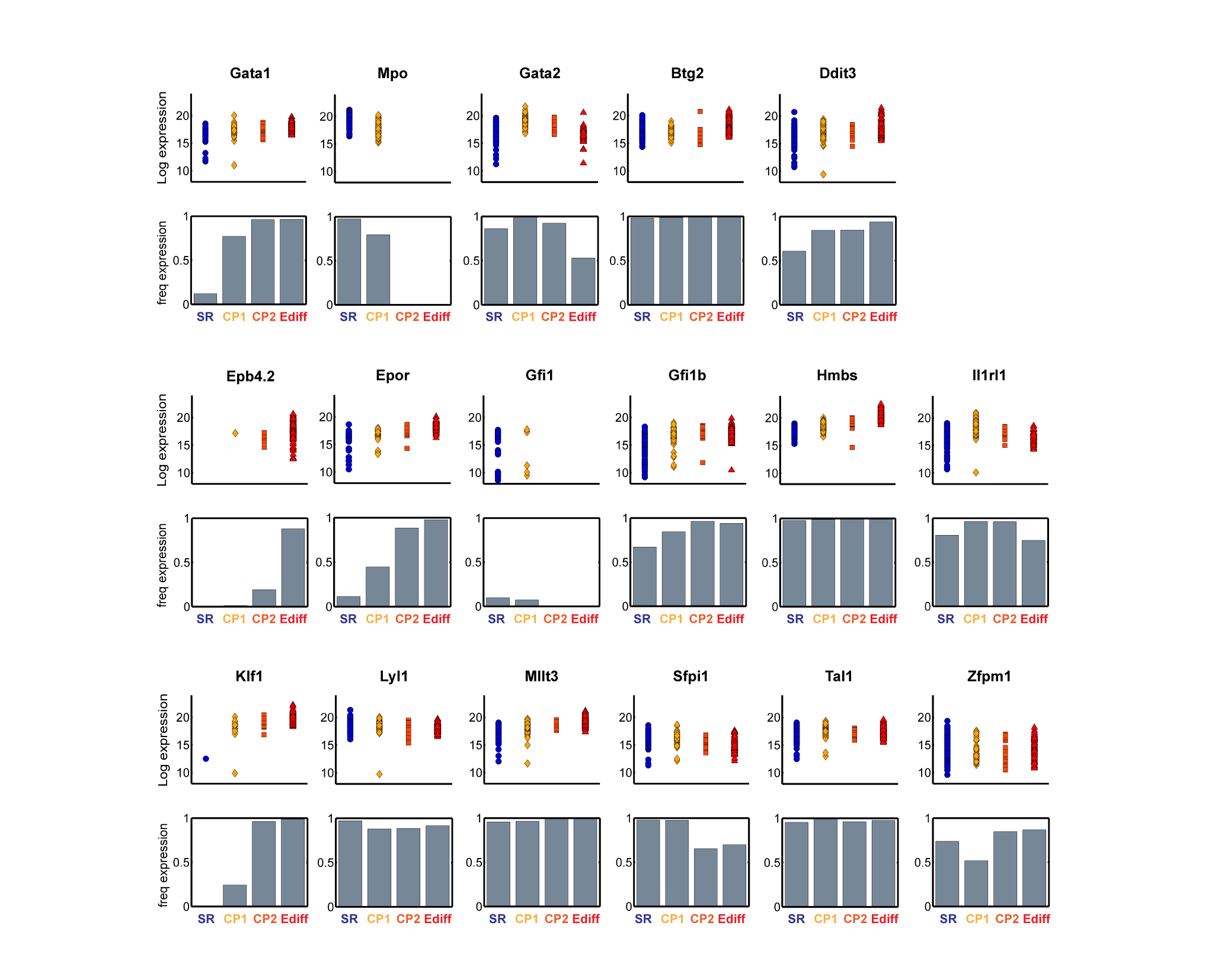

Supplement: Figure S2 — Single-cell gene expression profiles. Single cell level (top) and frequency of expression (bottom) in SR (blue circles), CP1 (yellow diamonds), CP2 (orange squares) and Ediff (red triangles) populations for all genes. Different expression patterns are observed from monotonic increase (e.g. Gata1) or decrease (e.g. Mpo), to non-monotonic behavior (e.g Gata2, Btg2), suggesting potential roles in different stages of lineage specification. (TIF) [file pcbi.1003197.s002.tif]
