# Supplementary material for: Transcriptional Regulation of Lineage Commitment - A Stochastic Model of Cell Fate Decisions
Source: PLoS Comput Biol. 2013 Aug 22;9(8):e1003197. doi: 10.1371/journal.pcbi.1003197 (PMC3749951; doi:10.1371/journal.pcbi.1003197)
Supplement: Table S1 — Correlation analysis: SR population. Significant pairwise correlations between all genes in the SR population. For each pairwise comparison where at least 10 cells co-expressed both genes, Spearman correlation coefficient was considered significant for values above 0.3 at a 99% significance level (bold). (PDF) [file pcbi.1003197.s006.pdf]

	**Btg2**	**Ddit3**	**Epb4.2**	**Epor**	**Gata1**	**Gata2**	**Gfi1**	**Gfi1b**	**Hmbs**	**Il1rl1**	**Klf1**	**Lyl1**	**Mllt3**	**Mpo**	**Sfpi1**	**Tal1**	**Zfpm1**
**Btg2**	1	0	0	0	0	0	0	0	0	0	0	0	0	0	0	0	0
**Ddit3**	0,26	1	0	0	0	0	0	0	0	0	0	0	0	0	0	0	0
**Epb4.2**	-	-	-	0	0	0	0	0	0	0	0	0	0	0	0	0	0
**Epor**	**0,59**	0,32	-	1	0	0	0	0	0	0	0	0	0	0	0	0	0
**Gata1**	0,27	0,21	-	-	1	0	0	0	0	0	0	0	0	0	0	0	0
**Gata2**	0,26	0,15	-	0,06	0,13	1	0	0	0	0	0	0	0	0	0	0	0
**Gfi1**	0,21	0,24	-	-	-	0,30	1	0	0	0	0	0	0	0	0	0	0
**Gfi1b**	0,08	0,14	-	0,17	-0,07	0,08	0,24	1	0	0	0	0	0	0	0	0	0
**Hmbs**	0,05	0,13	-	-0,01	0,19	0,16	-0,32	0,19	1	0	0	0	0	0	0	0	0
**Il1rl1**	0,06	0,05	-	0,16	-0,10	0,24	-0,28	0,04	0,24	1	0	0	0	0	0	0	0
**Klf1**	-	-	-	-	-	-	-	-	-	-	-	0	0	0	0	0	0
**Lyl1**	0,13	0,14	-	0,22	0,11	0,29	-0,05	0,29	0,26	0,04	-	1	0	0	0	0	0
**Mllt3**	0,15	0,06	-	0,14	-0,21	0,23	0,19	0,18	0,22	0,23	-	0,21	1	0	0	0	0
**Mpo**	0,16	0,12	-	0,28	0,04	-0,01	0,30	0,12	0,13	-0,06	-	**0,31**	0,06	1	0	0	0
**Sfpi1**	0,27	0,06	-	0,09	0,11	-0,01	0,03	0,12	0,12	0,05	-	0,18	0,00	0,06	1	0	0
**Tal1**	0,20	0,14	-	0,25	0,06	0,28	0,42	0,01	0,08	0,23	-	0,03	0,17	-0,04	0,16	1	0
**Zfpm1**	0,03	-0,02	-	0,24	0,41	-0,07	0,07	-0,17	-0,26	0,17	-	-0,15	0,01	0,08	-0,09	0,08	1
