# Supplementary material for: Transcriptional Regulation of Lineage Commitment - A Stochastic Model of Cell Fate Decisions
Source: PLoS Comput Biol. 2013 Aug 22;9(8):e1003197. doi: 10.1371/journal.pcbi.1003197 (PMC3749951; doi:10.1371/journal.pcbi.1003197)
Supplement: Table S2 — Correlation analysis: CP1 population. Significant pairwise correlations between all genes in the CP1 population. For each pairwise comparison where at least 10 cells co-expressed both genes, Spearman correlation coefficient was considered significant for values above 0.3 at a 99% significance level (bold). (PDF) [file pcbi.1003197.s007.pdf]

	**Btg2**	**Ddit3**	**Epb4.2**	**Epor**	**Gata1**	**Gata2**	**Gfi1**	**Gfi1b**	**Hmbs**	**Il1rl1**	**Klf1**	**Lyl1**	**Mllt3**	**Mpo**	**Sfpi1**	**Tal1**	**Zfpm1**
**Btg2**	1	0	0	0	0	0	0	0	0	0	0	0	0	0	0	0	0
**Ddit3**	**0,32**	1	0	0	0	0	0	0	0	0	0	0	0	0	0	0	0
**Epb4.2**	-	-	-	0	0	0	0	0	0	0	0	0	0	0	0	0	0
**Epor**	0,00	-0,14	-	1	0	0	0	0	0	0	0	0	0	0	0	0	0
**Gata1**	0,07	0,20	-	0,05	1	0	0	0	0	0	0	0	0	0	0	0	0
**Gata2**	0,26	**0,36**	-	0,02	0,14	1	0	0	0	0	0	0	0	0	0	0	0
**Gfi1**	-	-	-	-	-	-	-	0	0	0	0	0	0	0	0	0	0
**Gfi1b**	**0,31**	**0,33**	-	0,00	**0,37**	0,15	-	1	0	0	0	0	0	0	0	0	0
**Hmbs**	0,11	**0,35**	-	0,23	0,25	-0,04	-	0,25	1	0	0	0	0	0	0	0	0
**Il1rl1**	**0,41**	**0,35**	-	0,05	-0,11	**0,48**	-	0,26	0,18	1	0	0	0	0	0	0	0
**Klf1**	0,17	-0,05	-	0,69	0,35	-0,21	-	**0,66**	0,48	0,00	1	0	0	0	0	0	0
**Lyl1**	0,16	0,16	-	-0,18	0,10	0,13	-	0,20	-0,21	0,13	0,30	1	0	0	0	0	0
**Mllt3**	0,19	**0,39**	-	0,24	0,26	0,20	-	**0,34**	**0,46**	0,24	0,07	-0,13	1	0	0	0	0
**Mpo**	0,02	-0,17	-	-0,24	-0,10	-0,23	-	-0,12	-0,30	-0,07	-0,21	0,22	-0,27	1	0	0	0
**Sfpi1**	0,28	0,07	-	-0,15	0,04	0,16	-	0,11	-0,03	0,21	-0,09	**0,37**	0,06	0,09	1	0	0
**Tal1**	**0,36**	0,22	-	-0,26	0,00	0,23	-	0,23	0,12	0,21	-0,10	0,12	0,14	0,04	0,02	1	0
**Zfpm1**	0,06	0,24	-	0,09	0,06	0,02	-	0,29	0,28	0,13	-0,18	-0,01	0,30	-0,16	0,02	-0,16	1
