# Supplementary material for: Transcriptional Regulation of Lineage Commitment - A Stochastic Model of Cell Fate Decisions
Source: PLoS Comput Biol. 2013 Aug 22;9(8):e1003197. doi: 10.1371/journal.pcbi.1003197 (PMC3749951; doi:10.1371/journal.pcbi.1003197)
Supplement: Table S3 — Correlation analysis: Ediff population. Significant pairwise correlations between all genes in the Ediff population. For each pairwise comparison where at least 10 cells co-expressed both genes, Spearman correlation coefficient was considered significant for values above 0.3 at a 99% significance level (bold). (PDF) [file pcbi.1003197.s008.pdf]

	**Btg2**	**Ddit3**	**Epb4.2**	**Epor**	**Gata1**	**Gata2**	**Gfi1**	**Gfi1b**	**Hmbs**	**Il1rl1**	**Klf1**	**Lyl1**	**Mllt3**	**Mpo**	**Sfpi1**	**Tal1**	**Zfpm1**
**Btg2**	1	0	0	0	0	0	0	0	0	0	0	0	0	0	0	0	0
**Ddit3**	**0,75**	1	0	0	0	0	0	0	0	0	0	0	0	0	0	0	0
**Epb4.2**	**0,72**	**0,65**	1	0	0	0	0	0	0	0	0	0	0	0	0	0	0
**Epor**	**0,63**	**0,55**	**0,50**	1	0	0	0	0	0	0	0	0	0	0	0	0	0
**Gata1**	**0,50**	**0,44**	**0,43**	**0,47**	1	0	0	0	0	0	0	0	0	0	0	0	0
**Gata2**	0,25	0,32	0,36	0,25	0,16	1	0	0	0	0	0	0	0	0	0	0	0
**Gfi1**	-	-	-	-	-	-	-	0	0	0	0	0	0	0	0	0	0
**Gfi1b**	0,27	**0,37**	**0,38**	0,25	0,10	**0,41**	-	1	0	0	0	0	0	0	0	0	0
**Hmbs**	**0,81**	**0,66**	**0,66**	**0,73**	**0,46**	-0,11	-	0,28	1	0	0	0	0	0	0	0	0
**Il1rl1**	0,21	**0,48**	0,31	0,06	0,05	**0,52**	-	**0,55**	-0,01	1	0	0	0	0	0	0	0
**Klf1**	**0,63**	**0,64**	**0,67**	**0,64**	**0,32**	**0,50**	-	**0,57**	**0,60**	**0,60**	1	0	0	0	0	0	0
**Lyl1**	**0,54**	**0,55**	**0,59**	**0,38**	0,27	0,25	-	**0,49**	**0,45**	**0,42**	**0,49**	1	0	0	0	0	0
**Mllt3**	**0,69**	**0,59**	**0,63**	**0,50**	**0,36**	**0,39**	-	**0,43**	**0,65**	**0,36**	**0,72**	**0,64**	1	0	0	0	0
**Mpo**	-	-	-	-	-	-	-	-	-	-	-	-	-	-	0	0	0
**Sfpi1**	**0,53**	**0,67**	**0,66**	**0,38**	0,22	0,43	-	**0,45**	**0,38**	**0,68**	**0,75**	**0,45**	**0,57**	-	1	0	0
**Tal1**	**0,52**	**0,57**	**0,55**	**0,57**	**0,40**	0,38	-	**0,41**	**0,58**	**0,38**	**0,65**	0,25	**0,44**	-	**0,48**	1	0
**Zfpm1**	0,20	**0,34**	0,23	0,11	-0,05	0,12	-	0,22	0,14	**0,35**	**0,35**	0,16	0,21	-	**0,46**	0,10	1
