# Supplementary material for: Transcriptional Regulation of Lineage Commitment - A Stochastic Model of Cell Fate Decisions
Source: PLoS Comput Biol. 2013 Aug 22;9(8):e1003197. doi: 10.1371/journal.pcbi.1003197 (PMC3749951; doi:10.1371/journal.pcbi.1003197)
Supplement: Table S4 — Parameter values for the random telegraph model of transcriptional bursting ( Figure 3A ). Except for , which was obtained from the literature (see Main Text), all parameters were obtained from fitting to experimental SR expression distributions for each gene. (PDF) [file pcbi.1003197.s009.pdf]

	Gata1	Gata2	Mpo
τ_{ON} (h)	72.6	2.80	1.19
τ_{OFF} (h)	3.65	1.52	5.16
τ_{RNA} (h)	0.257	0.128	1.13
τ_{D} (h)	2.86	2.14	5.71
ξ	16.01	16.08	19.31
