# Supplementary material for: Transcriptional Regulation of Lineage Commitment - A Stochastic Model of Cell Fate Decisions
Source: PLoS Comput Biol. 2013 Aug 22;9(8):e1003197. doi: 10.1371/journal.pcbi.1003197 (PMC3749951; doi:10.1371/journal.pcbi.1003197)
Supplement: Text S1 — Gene expression data, cell culture data and Gata1 perturbation experiments. The single cell gene expression and clonal culture-reconstitution experiments from [21] are summarized. Details from the Gata1-ERT perturbation experiment are given. (PDF) [file pcbi.1003197.s010.pdf]

Supplementary Text S1

Transcriptional regulation of lineage commitment – a stochastic model of cell fate decisions

J. Teles, C. Pina, P. Edén, M. Ohlsson, T. Enver and C. Peterson

A. Gene expression data

Single-cell quantitative RT-qPCR data for 17 genes (*Btg2, Ddit3, Epb4.2, Epor, Gata1, Gata2, Gfi1, Gfi1b, Hmbs, Il1rl1, Klf1, Lyl1, Mllt3, Mpo, Sfpi1, Tal1, Zfpm1*) in 319 self-renewing (SR), 109 erythroid-committed (CP) and 42 erythroid-differentiated (Ediff) EML cells were directly obtained from a recent publication [1]. The data were generated using a Fluidigm platform, as described therein. SR cells were CD34+Itgae-, CD34+Sca1lo and CD34+Sca1hi; CP cells were CD34-Sca1lo and CD34-Itgae-; Ediff cells were kit-. An additional set of 41 Ediff cells isolated as kit- cells after a 6-day differentiation culture seeded with CD34+ Itgae- SR cells was also included. Culture conditions and cell sorting strategies were as described in [1]. The 17 genes were present in all datasets in [1]; the data also included 4 control genes (*Atp5a1, B2m, Hprt1, Ubc*) that were used for filtering and normalization purposes, as detailed in the same publication.

B. Cell culture data

Clonal culture-reconstitution experiments were presented and described in Pina et al. Briefly, single cells prospectively isolated as SR (CD34+Itgae-) or CP (CD34-Itgae-) were seeded into the wells of a 96-well plate containing self-renewing EML culture medium; at regular intervals, each well was visually inspected and live and dead cell counts recorded. For the purposes of this study, we used live and dead cell counts for days 1 and 2 of culture for a total of 96 wells per cell fraction. In population-based reconstitution experiments, prospectively isolated CD34+Itgae- SR cells were cultured in bulk in self-renewing EML culture medium; at regular intervals, live and dead cell counts were taken, and a culture sample analyzed by flow cytometry for CD34 and Itgae surface antigen detection. Culture conditions and flow cytometry protocols and antibodies used are detailed in [1]. For the purposes of this study, data from day 2 of culture were considered.

C. Gata1 perturbation experiments

Bulk EML cultures were transduced under maintenance culture conditions with lentiviruses encoding a GATA1-ERT fusion construct [2, 3] or the respective empty vector control. After a 6-hour transduction, cells were extensively washed and cultured for 2 days prior to enrichment for GFP+-transduced cells by cell sorting. Two days later, the self-renewing fraction

was sorted as CD34+CD103-Sca1lo GFP+ [1], allowed to recover in maintenance culture conditions for 3 hours, and activated for 16h with 2M of 4-hydroxy-tamoxifen. At the end of the activation period, cells were extensively washed and single GFP+ cells were deposited onto the wells of a 96-well plate containing 100l/well of EML maintenance culture medium. Clonal growth was followed for 8 days with individual wells scored for live cell numbers at regular intervals [1]. Wells containing $>$ 100 cells at day 8 were scored as containing the progeny of an SR cell.

[1] Pina C, Fugazza C, Tipping AJ, Brown J, Soneji S, et al. (2012) Inferring rules of lineage commitment in haematopoiesis. Nature cell biology 14: 287–94.

[2] Heyworth C, Gale K, Dexter M, May G, Enver T (1999) A gata-2/estrogen receptor chimera functions as a ligand-dependent negative regulator of self-renewal. Genes and Development 13: 1847-1860.

[3] Heyworth C, Pearson S, May G, Enver T (2002) Transcription factor-mediated lineage switching reveals plasticity in primary committed progenitor cells. The EMBO journal 21: 3770–81.
